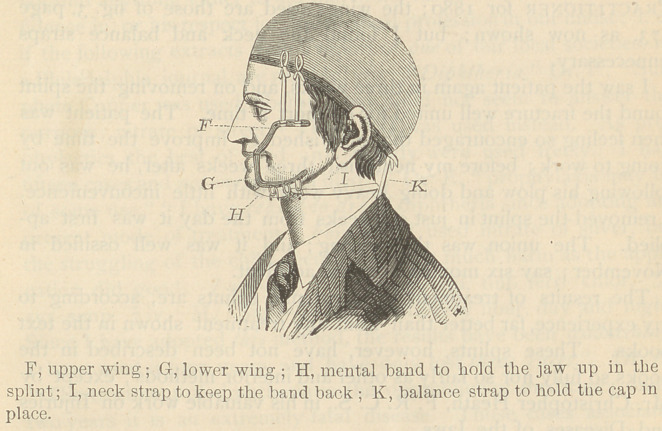# Treatment of Fractured Jaw without Teeth in the Mouth

**Published:** 1881-03

**Authors:** J. Adams Bishop

**Affiliations:** New York


ARTICLE IV.

### TREATMENT OF FRACTURED JAW WITHOUT TEETH IN THE MOUTH.

BY J. ADAMS BISHOP, M. D., NEW YORK.

I speak with diffidence on a subject which has been so ably presented by my friend, Dr. Gunning, in your Journal for 1880, but as he refers to a novel case treated by me, I venture to show your readers some of its difficulties, and even with the few facilities at command, the very favorable result of the treatment. When on a visit in May, 1879, I was called by Dr Phinney, of Norwich, Conn., to see the fracture; and it was decided to treat it with an interdental splint.

The patient, S. B., aged seventy years, while recovering from a severe attack of pneumonia, had tried to pass from one floor of his house to the other, but being weak fell down the stairs and received a compound fracture of the lower jaw, the bone protruding through the gum. His last tooth, a canine on the right side, was knocked out and lost. The fracture was ten days old with considerable displacement; Dr. Phinney had reduced it with much care and skill, but could not keep the parts in position. There being an entire absence of teeth in the mouth; my first difficulty was to secure the fragments in position. I had never met with such a case; such a one seems never to have presented itself to Dr. Gunning in more than forty years' experience, and M. Malgaigne, in 1847 says, no case of lower jaw fractured when destitute of teeth, seems to have been presented for treatment. I was without cups or proper conveniences for taking the impressions, and in the country where no Dental office was near. Having, however, a little plaster with a pair shears and plyers, I made cups for upper and lower jaw out of a tin milk-pan, and succeeded in getting good impressions from which I got up the splint at my office in New York. I did not see the patient again till six days after; but with a little trouble the fractured jaw was placed in the splint.

I was pleased to notice the confidence which the patient expressed when the splint was put on. He at once felt that his jaw was secured from the displacing action of the muscles; and he was free from pain.

He could breathe better and swallow with greater ease. I have found this so with many of the patients I have seen under this mode of treatment. Dr. Phinney, who was present, took charge of the patient.

F, upper wing; G, lower wing; H, mental band to hold the jaw up in the splint; I, neck strap to keep the band back; K, balance strap to hold the cap in place.

The difficulty of holding the fractured ends in contact, arose from the absence of teeth; had only one remained in each fragment of the jaw a hard rubber splint could have been fitted to the gum all around and secured by ligatures or screws to a tooth on each side of the fracture. This would have held the broken ends together without restricting the natural movements of the jaw in eating and talking. Further, the alveolar of the lower jaw having been absorbed through the loss of the teeth, the gum slanted down from the ascending *rami* to the front, while the outside of the jaw always tapers from the angles to the chin, and another slant passed from the throat up and out to the same point; thus the three surfaces all tapered. Therefore a bandage, even if fastened to wings projecting from the splint through each corner of the mouth, would not hold the jaw in its place. It was therefore necessary to make a resting place for the broken bone by means of the upper jaw, consequently, the upper surface of the splint was made to fit against the upper gum. Then by means of the wings a cap on the head held the splint securely, and a strip of muslin passed from one lower wing to the other, under the chin, thus holding the fragments of the jaw in place. The splint had an opening in front, through which to speak and eat, as seen in fig. 2, page 572, of the INDEPENDENT PRACTITIONER for 1880; the wings used are those of fig. 3, page 573, as now shown; but I found the neck and balance straps unnecessary.

I saw the patient again in three weeks, and on removing the splint found the fracture well united for so short a time. The patient was then feeling so encouraged that he wished to improve the time by going to work; before my next visit, three weeks after, he was out following his plow and doing heavy work with little inconvenience. I removed the splint in just six weeks from the day it was first applied. The union was then strong; and it was well ossified in November; say six months from the accident.

The results of treatment by interdental splints are, according to my experience, far better than from any treatment shown in the text books. These splints, however, have not been described in the books so fully nor so fairly as other and inferior methods; except by Mr. Christopher Heath, F. R. C. S., in his valuable work on Injuries and Diseases of the Jaws.

## Figures and Tables

**Figure f1:**